# Engaging innate immunity for targeting the epidermal growth factor receptor: Therapeutic options leveraging innate immunity versus adaptive immunity versus inhibition of signaling

**DOI:** 10.3389/fonc.2022.892212

**Published:** 2022-09-14

**Authors:** Gabriele Hintzen, Holger J. Dulat, Erich Rajkovic

**Affiliations:** Research and Development, Affimed GmbH, Heidelberg, Germany

**Keywords:** epidermal growth factor receptor, innate immunity, adaptive immunity, cancer, tyrosine kinase inhibitor, bispecific antibody, CAR-T therapy, innate cell engager

## Abstract

The epidermal growth factor receptor (EGFR) is a key player in the normal tissue physiology and the pathology of cancer. Therapeutic approaches have now been developed to target oncogenic genetic aberrations of EGFR, found in a subset of tumors, and to take advantage of overexpression of EGFR in tumors. The development of small-molecule inhibitors and anti-EGFR antibodies targeting EGFR activation have resulted in effective but limited treatment options for patients with mutated or wild-type EGFR-expressing cancers, while therapeutic approaches that deploy effectors of the adaptive or innate immune system are still undergoing development. This review discusses EGFR-targeting therapies acting through distinct molecular mechanisms to destroy EGFR-expressing cancer cells. The focus is on the successes and limitations of therapies targeting the activation of EGFR versus those that exploit the cytotoxic T cells and innate immune cells to target EGFR-expressing cancer cells. Moreover, we discuss alternative approaches that may have the potential to overcome limitations of current therapies; in particular the innate cell engagers are discussed. Furthermore, this review highlights the potential to combine innate cell engagers with immunotherapies, to maximize their effectiveness, or with unspecific cell therapies, to convert them into tumor-specific agents.

## Expression and function of the epidermal growth factor receptor in normal and tumor tissue

Epidermal growth factor receptor (EGFR, also known as ErbB1/HER1) is a cell-surface receptor tyrosine kinase that belongs to the ErbB family of receptors composed of four closely related members: EGFR (ErbB1/HER1), ErbB2 (HER2), ErbB3 (HER3) and ErbB4 (HER4) (1). Under normal physiological conditions, EGFR is expressed in nearly all cell types and tissues, with the exception of hematopoietic lineage cells, and those in bone marrow, spleen, soft tissues, adrenal gland and specific brain tissues, where the EGFR protein is undetectable (2).

EGFR exerts multifaceted functions in the maintenance of normal epithelial tissue homeostasis by driving cell proliferation, growth, differentiation, migration, and survival through a ligand-dependent activation of its kinase activity, required for the initiation of multiple signaling pathways within the cell (3, 4). The binding of ligands such as epidermal growth factor (EGF), amphiregulin, epiregulin, transforming growth factor-α and others to the extracellular domain of EGFR leads to the formation of EGFR homodimers or heterodimers with the ErbB2, ErbB3 or ErbB4 receptors, the activation of the kinase activity and the transphosphorylation of the key tyrosine residues in the intracellular kinase domain and the C-terminal tail (1). These phosphorylated residues act as a scaffold for the binding of numerous signaling proteins, which initiate the RAS-RAF-MEK-ERK, AKT-PI3K, PLCγ1-PKC, JNK, and JAK-STAT3 signaling pathways (3). Moreover, it has been shown that EGFR dimers with a perturbed catalytic activity can also sustain cell survival signals using the kinase activity-independent scaffolding function (5, 6).

Considering the fundamental role of EGFR in maintaining the homeostasis of healthy tissue, it is not surprising that *EGFR* gain-of-function mutations are often detected in some tumor types. *EGFR* activating mutations in the tyrosine kinase domain leading to the ligand-independent activation of EGFR are frequently detected in non-small cell lung cancer (NSCLC) and glioblastoma but are rarely found in other tumor types (7–11), providing a possible foundation for the tumor type-specific responses to EGFR-targeted therapies and immunotherapies. The frequency of *EGFR*-activating mutations in tumor tissue also varies among different global demographics, with 30–40% of patients with NSCLC from East Asia exhibiting these mutations, but only 5–15% of patients of non-Asian origin (7, 12). In glioblastoma, *EGFR* aberrations frequently found also include mutations and various deletions in the extracellular domain of EGFR, with the truncated variant EGFRvIII, which lacks exons 2–7, being the most common deletion (13). Interestingly, although the EGFRvIII variant does not require ligand for its activation, it has a relatively weak constitutive kinase activity (8, 13) and the resulting growth advantage is believed to be conferred by an impaired endocytic pathway preventing physiological downregulation of the receptor (14).

In cancers that may not necessarily show high rates of *EGFR*-activating mutations, deregulation can also occur through overexpression. Numerous studies have reported overexpression of EGFR in 27–100% of solid tumors, including NSCLC, colorectal cancer (CRC), squamous cell carcinoma of the head and neck (SCCHN), gastric-gastroesophageal junction cancer, urothelial cancer, clear cell renal cell carcinoma, hepatocellular carcinoma, glioblastoma, pancreatic cancer, and breast cancer, among others (4, 6, 10, 15). Overexpression of EGFR may result from an *EGFR* copy number gain due to amplification of the genomic region comprising the *EGFR* gene locus (16–19) and can lead to increased capability to form ligand-independent EGFR homodimers and heterodimers (20). In particular EGFR/ErbB2 heterocomplexes show a strong ligand-independent constitutive activity, resistance to the ubiquitin-dependent degradation of heterodimers and high levels of persistent signaling (21). In several cancer types, EGFR expression levels correlate with the disease prognosis (15). In patients with head and neck, ovarian, cervical, bladder and esophageal cancers, elevated EGFR levels were found to be a strong prognostic factor and were associated with reduced recurrence-free or overall survival (15), while in patients with gastric, breast, endometrial and colorectal cancers, increased EGFR levels correlated with poor survival rates, but were considered a modest prognostic factor (15).

## EGFR-targeting therapeutic approaches

The gain-of-function alterations in *EGFR*, such as substitution mutations, deletions and insertions, and high levels of cell-surface EGFR expression have been proven to have a crucial role in sustaining cancer cell proliferation, growth and survival, and cancer progression (12, 15). This, without doubt, has highlighted EGFR as an attractive therapeutic target for the treatment of patients with EGFR-expressing cancer.

Broadly, there are two main types of therapies approved to target EGFR: small molecule tyrosine kinase inhibitors (TKIs) and anti-EGFR antibodies. TKIs act to inhibit EGFR kinase activity, thus attenuating downstream signal transduction (22); whereas anti-EGFR antibodies serve to block ligand binding to the extracellular portion of EGFR leading to inhibition of downstream signaling, but also have the capacity to leverage cytotoxic immune cells to induce anti-tumor antibody-dependent cellular cytotoxicity (ADCC) (23, 24). Both types of therapy have demonstrated efficacy in subsets of patients with certain tumor types, with TKIs exhibiting efficacy in, for example, NSCLC with *EGFR*-activating mutations (25), and anti-EGFR antibodies in tumor types such as metastatic CRC (mCRC) (9), and SCCHN (26). Despite the effectiveness of these agents, however, several drawbacks have emerged, such as the development of drug resistance (26, 27). As such, there remains a significant unmet need in EGFR-expressing tumors for therapies which induce long-term remissions.

Promising novel therapeutic approaches exploit the frequent overexpression of EGFR in a broad range of different cancers and the tumor immune microenvironment. A number of therapeutic agents including CAR-T cells, CAR-NK cells, bispecific T-cell engagers, and bispecific innate cell engagers – all of which are currently being investigated in preclinical and clinical studies - exploit approaches to harness effectors of the adaptive or innate immune system in order to bridge them with the tumor cell-surface EGFR and prime them for destruction of the EGFR-expressing cancer cells (28, 29).

## Therapeutic agents inhibiting EGFR activation and signaling

### Small-molecule tyrosine kinase inhibitors

**Figure 1:**
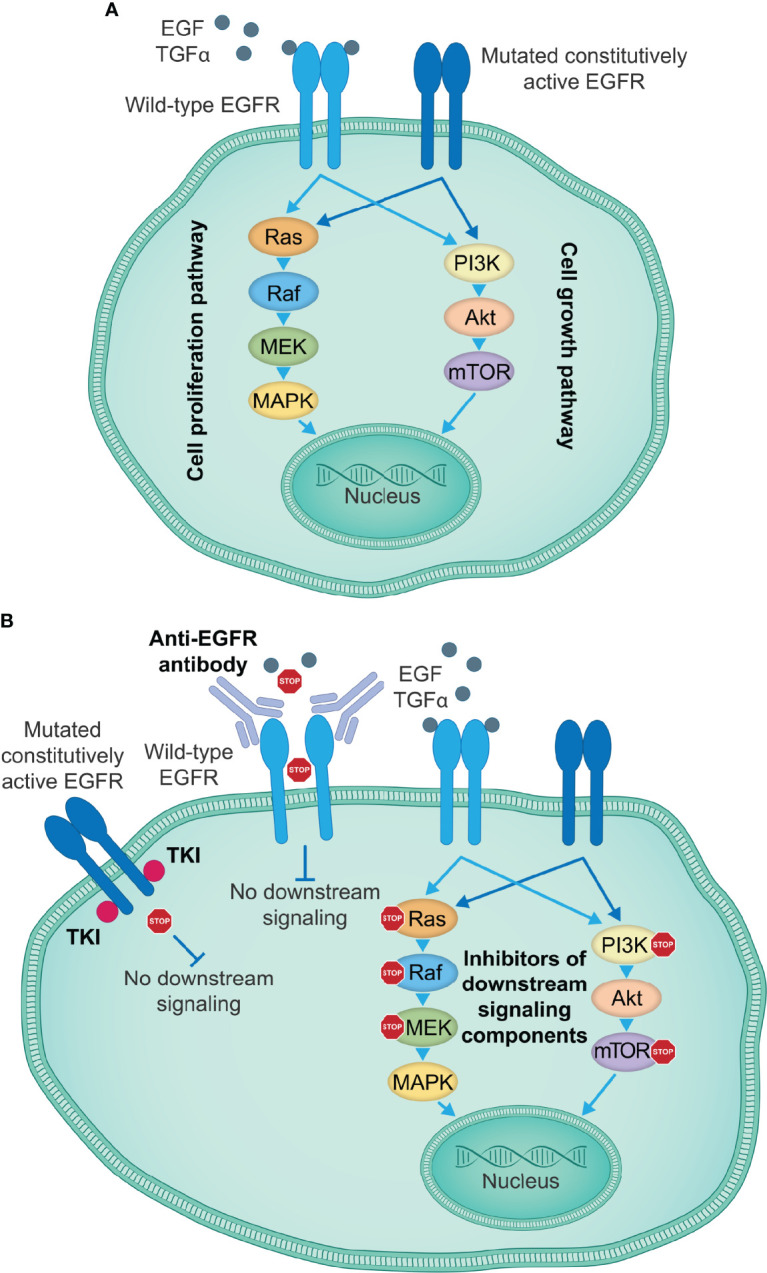
EGFR-targeting therapies inhibiting EGFR activation and signal transduction. **(A)** EGFR signaling induced by specific ligands, including EGF and TGFα among others, starts with the conformational switch upon binding of a ligand to the EGFR extracellular domain, the dimerization of EGFR monomers and transphosphorylation of intracellular tyrosine kinase domains, which creates docking sites for the adaptor molecules, leading to the activation of key downstream signaling pathways that govern cell proliferation and growth. Certain mutations in the EGFR kinase or extracellular domain induce a constitutively active state and ligand-independent oncogenic signaling downstream of activated EGFR, thus causing uncontrolled tumor cell proliferation and growth. **(B)** Currently approved therapeutic agents for the treatment of patients with lung or colorectal cancer, or SCCHN include TKIs targeting the EGFR kinase activity (e.g., first-generation small-molecule EGFR kinase inhibitors gefitinib, erlotinib and icotinib), which show particularly high efficacy in the presence of activating EGFR kinase domain mutations, and anti-EGFR antibodies (e.g., cetuximab) that prevent binding of a ligand to the wild-type EGFR and the activation of EGFR and the downstream signaling cascades. Other therapeutic approaches take advantage of individual signaling components downstream of EGFR to disrupt the EGFR signaling cascades and impair tumor cell proliferation and growth. EGF, epidermal growth factor; EGFR, EGF receptor; SCCHN, squamous cell carcinoma of the head and neck; TGFα, transforming growth factor α; TKIs, tyrosine kinase inhibitors.

EGFR TKIs are orally available ATP-competitive compounds that reversibly or irreversibly bind the ATP-binding site in the EGFR tyrosine kinase domain, thus preventing EGFR activation, transphosphorylation of tyrosine residues and transduction of downstream signaling pathways (**Figure 1**). With the exception of erlotinib, TKIs targeting EGFR have only been approved by the U.S. Food and Drug Administration (FDA) for the treatment of patients with NSCLC, whose tumors frequently have activating *EGFR* mutations; several generations of these are available for clinical use (30) (**Table 1**). In contrast, TKIs have shown limited efficacy in tumors where mutations in *EGFR* are not present, such as NSCLC with wild-type *EGFR* (102), and where *EGFR*-activating mutations are less common, such as in mCRC and SCCHN (9–11, 103, 104). As such, EGFR TKIs will mostly be discussed below in the context of NSCLC with *EGFR*-activating mutations. Clinical efficacy data from key randomized trials mentioned below are given in **Table 1**.

**Table 1:** Clinically approved inhibitors of EGFR signaling, acquired resistance mechanisms, clinical efficacy, and safety profile identified in patients with cancer.

Drug	Approved Indications^a^	Key Acquired Resistance Mechanisms in Clinical Setting	Efficacy Data from Key Phase III Clinical Trials	Most Common AEs (All Grade)
Erlotinib (first-generation) (31–39)	NSCLC: First-line, maintenance or second and greater-line treatment after failure of ≥1 chemotherapy regimen of pts with mNSCLC and *EGFR* exon 19 deletions or L858R mutation Metastatic pancreatic cancer: in combination with gemcitabine	EGFR T790M mutation *ERBB2* amplification *MET* amplification	NSCLC: OPTIMAL (erlotinib vs. CT) – ORR: 83% vs. 36% (p<0.0001); PFS: 13.1 months vs. 4.6 months (p<0.0001); OS: 22.8 months vs. 27.2 months (35, 39) EURTAC (erlotinib vs. CT) – ORR: 64% vs. 18% (p<0.0001); PFS: 9.7 months vs. 5.2 months (p<0.0001); OS: 19.3 months vs. 19.5 months (36) ENSURE (erlotinib vs. CT) – ORR: 62.7% vs. 33.6%; PFS: 11.0 months vs. 5.6 months (p<0.0001); OS: 26.3 months vs. 25.5 months (37) Metastatic pancreatic cancer: NCIC CTG PA.3 (erlotinib plus gemcitabine vs. gemcitabine) – ORR: 8.6% vs. 8.0%; PFS: 3.75 months vs. 3.55 months (p=0.004); OS: 6.24 months vs. 5.91 months (p=0.038) (38)	Rash, edema, diarrhea, anorexia, fatigue, dyspnea, cough, nausea, infection, vomiting, pyrexia, and decreased weight
Gefitinib (first-generation) (33, 34, 40–45)	First-line treatment of pts with mNSCLC/*EGFR* exon 19 deletions or L858R mutation	EGFR T790M mutation *ERBB2* amplification *MET* amplification	IPASS (gefitinib vs. CT) – ORR: 43.0% vs. 32.2% (p<0.001); PFS: 5.7 months vs. 5.8 months; OS: 18.6 months vs. 17.3 months (42) WJTOG-3405 (gefitinib vs. CT) – ORR: 62.1% vs. 32.2% (p<0.0001); PFS: 9.2 months vs. 6.3 months (p<0.0001); OS: 34.9 months vs. 37.3 months (43, 45) NEJ002 (gefitinib vs. CT) – ORR: 73.7% vs. 30.7% (p<0.001); PFS: 10.8 months vs. 5.4 months (p<0.001); OS: 30.5 months vs. 23.6 months (44)	Skin reactions, diarrhea, ALT increased, AST increased, and proteinuria
Icotinib (46, 47)	First-line treatment of pts with mNSCLC and non-resistant *EGFR* mutations; and those who progress after platinum-based chemotherapy	EGFR T790M mutation	ICOGEN (icotinib vs. gefitinib) – ORR: 27.6% vs. 27.2%; PFS: 4.6 months vs. 3.4 months; OS: 13.3 months vs. 13.9 months (46) CONVINCE (icotinib vs. CT) – ORR: NR; PFS: 11.2 months vs. 7.9 months (p=0.006); OS: 30.5 months vs. 32.1 months (47)	Rash, diarrhea, increased ALT, increased AST, leukopenia
Afatinib (second-generation) (48–54)	First-line treatment of pts with mNSCLC and non-resistant *EGFR* mutations; and those who progress after platinum-based chemotherapy	EGFR T790M mutation	LUX-Lung 3 (afatinib vs. CT) – ORR: 56% vs. 23% (p=0.001); PFS: 11.1 months vs. 6.9 months (p=0.001); OS: *Ex19del =* 33.3 months vs. 21.1 months (p=0.0015), *L858R* = 27.6 months vs. 40.3 months, whole population = 31.6 months vs. 28.2 months (52, 54) LUX-Lung 6 (afatinib vs. CT) – ORR: 66.9% vs. 23.0% (p<0.0001); PFS: 11.0 months vs. 5.6 months (p<0.0001); OS: *Ex19del =* 31.4 months vs. 18.4 months (p=0.023), *L858R* = 19.6 months vs. 24.3 months, whole population = 23.6 months vs. 23.5 months (53, 54)	Rash/acneiform dermatitis, pruritus, diarrhea, stomatitis, infections, decreased appetite, increased ALT, and increased AST
Dacomitinib (second-generation) (55–59)	First-line treatment of pts with metastatic NSCLC with *EGFR* exon 19 deletion or the L858R substitution mutation	EGFR T790M mutation	ARCHER 1009 (dacomitinib vs. erlotinib) – ORR: 11% vs. 8%; PFS: 2.6 months vs. 2.6 months; OS: 7.9 months vs. 8.3 months (57) ARCHER 1050 (dacomitinib vs. gefitinib) – ORR: 75% vs. 72%; PFS: 14.7 months vs. 9.2 months (p<0.0001); 34.1 months vs. 27.0 months (p=0.0155) (58, 59)	Diarrhea, stomatitis, rash, paronychia, dry skin, alopecia, pruritus, decreased appetite, decreased weight, cough, anemia, lymphopenia, hypoalbuminemia, increased ALT, increased AST, hyperglycemia, hypocalcemia, hypokalemia, hyponatremia, increased creatinine, increased AP, and hypomagnesemia
Osimertinib (third-generation) (60–70)	First-line treatment of pts with mNSCLC and *EGFR* exon 19 deletions or exon 21 L858R mutation; treatment of pts with EGFR T790M mutation-positive mNSCLC who progressed on or after EGFR TKI therapy	C797S mutation in the same allele with the T790M mutation EGFR L792F/H/Y, G796S/R, L718Q, L798I, L692V and E709K mutations, and exon 20 insertion Loss of T790M mutation Oncogenic gene fusions *MET*, *FGFR1*, *EGFR*, *ERBB2, MAPK1* amplification HGF, EGF overexpression IGFR upregulation *PIK3CA*, *BRAF*, *KRAS, CDKN2A, ALK, KIT, RB1* mutations Histologic transformation (EMT and SCLC)	AURA3 (osimertinib vs. CT) – ORR: 71% vs. 31% (p<0.001); PFS: 10.1 months vs. 4.4 months (p<0.001); OS: 26.8 months vs. 22.5 months (66–68) FLAURA (osimertinib vs. gefitinib) – ORR: 80% vs. 76%; PFS: 18.9 months vs. 10.2 months (p<0.001); OS: 38.6 months vs. 31.8 months (p=0.046) (69, 70)	Rash, dry skin, nail toxicity, diarrhea, stomatitis, fatigue, and decreased appetite
Brigatinib (71, 72)	Treatment in combination with cetuximab of adult pts with ALK-positive mNSCLC	NR	Retrospective analysis (brigatinib in combination with cetuximab vs. CT) – ORR: 60% vs. 10%; PFS: 14 months vs. 3 months; OS: NR (72)	Diarrhea, fatigue, nausea, vomiting, abdominal pain, rash, pruritus, cough, myalgia, hypertension, headache, vomiting, dyspnea, back pain, increased CPK, increased AST and ALT, increased lipase, hyperglycemia, increased amylase, decreased phosphorus, increased AP, increased creatine, increased potassium, increased calcium, decreased magnesium, decreased hemoglobin, and lymphocyte count decreased
Cetuximab (73–95)	EGFR-expressing *KRAS* wild-type mCRC: first-line treatment in combination with FOLFIRI; in combination with irinotecan when pts are refractory to irinotecan-based chemotherapy; and as a single agent in pts who have failed oxaliplatin- and irinotecan-based chemotherapy and who are intolerant to irinotecan SCCHN: in combination with radiation therapy for pts with locally or regionally advanced SCCHN; in combination with platinum-based chemotherapy for pts with recurrent locoregional or metastatic SCCHN; and for pts with recurrent or metastatic SCCHN who are intolerant to irinotecan	mCRC: *KRAS*, *NRAS, BRAF, AKT1* and *PIK3CA* mutations EGFR ectodomain mutations S468R, G465R, G441R/E and K443T *MET*, *KRAS, FGFR1* and *ERBB2* amplification SCCHN: EGFR ectodomain mutations G33S, N56K (patient-derived HNSCC cells) and G465R ERBB3 activation and ERBB2/ERBB3 dimerization (PDX models)	mCRC: CRYSTAL (cetuximab + CT vs. CT) – ORR: 46.9% vs. 38.7% (p=0.004); PFS: 8.9 months vs. 8 months (p=0.048); OS: 19.9 months vs. 18.6 months (88) TAILOR (cetuximab + CT vs. CT) – ORR: 61.1% vs. 39.5% (p<0.001); PFS: 9.2 months vs. 7.4 months (p=0.004); OS: 20.7 months vs. 17.8 months (p=0.02) (89) Cetuximab + CT vs. cetuximab monotherapy in pts refractory to CT – ORR: 22.9% vs. 10.8% (p=0.007); PFS: 4.1 months vs. 1.5 months (p<0.001); OS: 8.6 months vs. 6.9 months (90) NCT00079066 (cetuximab vs. BSC) – ORR: 8.0% vs. 0% (p<0.001); PFS: 1.9 months vs. 1.8 months (p<0.001); OS: 6.1 months vs. 4.6 months (p=0.005) (91) BEACON CRC (cetuximab + encorafenib + binimetinib [triplet] vs. cetuximab + encorafenib [doublet] vs. cetuximab + irinotecan or cetuximab + CT [control]) – ORR: 26% vs. 20% vs. 2% (both p<0.001 vs. control); PFS: 4.3 months vs. 4.2 vs. 1.5 months (both p<0.001 vs control); OS: 9.0 months vs. 8.4 vs. 5.4 months (both p<0.001 vs. control) (92) EPIC (cetuximab + irinotecan vs. irinotecan) – ORR: 16.4% vs. 4.2% (p<0.0001); PFS: 4.0 months vs. 2.6 months (p<0.0001); OS: 10.7 months vs. 10.0 months (93) SCCHN: EXTREME (cetuximab + CT vs. CT) – ORR: 36% vs. 20% (p<0.001); PFS: 5.6 months vs. 3.3 months (p<0.001); OS: 10.1 months vs. 7.4 months (p=0.04) (94) NCT00004227 (cetuximab + RT vs. RT) – ORR 74% vs. 64% (p=0.02); PFS:17.1 months vs. 12.4 months (p=0.006); OS: 49.0 months vs. 29.3 months (p=0.018) (95)	mCRC: Acneiform rash, diarrhea, stomatitis, constipation, vomiting, infection SCCHN: Acneiform rash,^b^ fever, nausea, diarrhea, infection
Panitumumab (83, 96–99)	Monotherapy in pts with EGFR-expressing *KRAS* wild-type mCRC with disease progression on or following fluoropyrimidine-, oxaliplatin-, and irinotecan-containing chemotherapy regimens	*KRAS* mutations EGFR ectodomain mutations G465R/E and S464L *MET* amplification	PARADIGM (panitumumab + CT vs. bevacizumab + CT in left-sided tumors) – ORR: 80.2% vs. 68.6%; PFS: 13.7 months vs. 13.2 months; OS: 37.9 months vs. 34.3 months (p=0.031) (96) NCT00113763 (panitumumab vs. BSC) – ORR: 10% vs. 0% (p<0.0001); PFS: 1.8 months vs. 1.7 months; OS at a median follow-up of 72 weeks: 19% vs. 16% (97) PRIME (panitumumab + CT vs. CT in pts with *wtKRAS*) – ORR: 55% vs. 48%; PFS: 9.6 months vs. 8.0 months (p=0.02); OS: 23.9 months vs. 19.7 months (98) PRIME (panitumumab + CT vs. CT in pts with mutant *KRAS*) – ORR: 40% vs. 40%; PFS: 7.3 months vs. 8.8 months (p=0.02); OS: 15.5 months vs. 19.3 months (98)	Erythema, pruritus, acneiform dermatitis, rash, skin fissures, dry skin, nausea, diarrhea, and hypomagnesemia
Necitumumab (100, 101)	First-line treatment for pts with mNSCLC in combination with gemcitabine or cisplatin	NR	SQUIRE (necitumumab + CT vs. CT) – ORR: 31% vs. 29%; PFS: 5.7 months vs. 5.5 months (p=0.02); OS: 11.5 months vs. 9.9 months (p=0.01) (101)	Rash and hypomagnesemia

a U.S. FDA approvals for patients with EGFR-aberrant cancer

b Includes acne, dermatitis acneiform, dry skin, exfoliative rash, rash, rash erythematous, rash macular, rash papular, and rash pustular

AEs, adverse events; ALK, anaplastic lymphoma kinase; ALT, alanine aminotransferase; AP, alkaline phosphatase; AST, aspartate aminotransferase; BRAF, *BRAF* proto-oncogene; CPK, creatine phosphokinase; EGF, epidermal growth factor; EGFR, epidermal growth factor receptor; EMT, epithelial-mesenchymal transition; ERBB2/3, v-erb-b2 erythroblastic leukemia viral oncogene homolog 2/3; FDA, Food and Drug Administration; FGFR, fibroblast growth factor receptor; FOLFIRI, folinic acid, fluorouracil, irinotecan; HGF, hepatocyte growth factor; IGFR, insulin growth factor receptor; KRAS, Kirsten rat sarcoma viral oncogene homolog; MAPK1, mitogen-activated protein kinase 1; mCRC, metastatic colorectal cancer; MET, mesenchymal-epithelial transition factor; mNSCLC, metastatic NSCLC; NR, not reported; NSCLC, non-small cell lung cancer; ORR, objective response rate; OS, overall survival; PDX, patient-derived xenografts; PFS, progression-free survival; PI3K, phosphoinositide 3-kinase; pts, patients; SCCHN, squamous cell carcinoma of the head and neck; SCLC, small cell lung carcinoma; TKI, tyrosine kinase inhibitor; wt, wild-type.

Erlotinib and gefitinib are two first-generation TKIs that bind reversibly to the active conformation of EGFR, blocking downstream signaling. Both are indicated for first-line use in patients with metastatic NSCLC carrying common *EGFR* sensitizing mutations in exons 18–21, such as the L858R missense mutation in exon 21 or exon 19 deletions, together accounting for more than 90% of exon 18–21 mutations in these patients (31, 105, 106). Erlotinib can also be indicated as a maintenance therapy or second- or subsequent-line therapy following disease progression and failure of at least one prior chemotherapy regimen (31). Several key phase III trials have demonstrated superior efficacy and progression-free survival (PFS) in patients treated with erlotinib or gefitinib versus standard chemotherapy regimens in patients with NSCLC exhibiting *EGFR*-sensitizing mutations, but no significant difference in overall survival (OS) was observed (33–37, 39, 42–45). These findings were confirmed by a meta-analysis of randomized trials comparing erlotinib/gefitinib monotherapy or the combination of erlotinib/gefitinib and chemotherapy with chemotherapy alone or placebo in patients with sensitizing *EGFR* mutation-positive-NSCLC, which showed a delayed disease progression when the treatment included gefitinib/erlotinib, but no effect on OS (107). Erlotinib is also indicated for the treatment of advanced or metastatic pancreatic cancer in combination with gemcitabine following a landmark phase III study, which demonstrated longer PFS and OS of the erlotinib combination versus gemcitabine alone (38). A third first-generation TKI, icotinib, is approved in China only for patients with advanced NSCLC carrying sensitizing *EGFR* mutations who have failed at least one prior chemotherapy regimen (108). The approval by the China National Medical Products Administration was based on the ICOGEN phase III study results that reported non-inferiority of icotinib versus gefitinib in terms of PFS and safety (46).

Despite proven efficacy of first-generation EGFR TKIs in subgroups of patients with *EGFR*-activating mutations in their tumors, resistance develops in most patients, with the median time to disease progression being around 12 months (34, 42, 109, 110). The underlying mechanism for acquired resistance is typically associated with the emergence of secondary *EGFR* mutations, that impair the binding of TKIs to EGFR, or alternatively, *via* mutations in other molecules that convey the EGFR-initiated signal transduction (34, 79, 109–111). The most common resistance mechanism, identified in approximately 50–70% of patients treated with first-generation TKIs, is the EGFR T790M mutation (32, 34, 41). The substitution of threonine with a much bulkier methionine leads to steric hindrance and the conformational change that prevents the binding of these TKIs to EGFR (112).

To improve TKI activity against common sensitizing *EGFR* mutations and the EGFR T790M resistance mutation, the second-generation irreversible EGFR inhibitors with a broader specificity to ErbB receptor family members have been developed (48, 55, 113). Preclinical studies demonstrated that this mode of action is more effective against EGFR sensitizing mutations and the EGFR T790M resistance mutation than inhibition by reversible first-generation EGFR inhibitors (114). Afatinib is a second-generation TKI that binds covalently and irreversibly to conserved cysteine residues in EGFR, ErbB2 and ErbB4 (48, 113). Afatinib is indicated for patients with NSCLC and exon 19 deletions (*Ex19del*), the L858R substitution mutation or other uncommon sensitizing *EGFR* mutations; it is also indicated for patients with squamous cell carcinoma of the lung after failure of first-line chemotherapy (48, 113). Key phase III trials have demonstrated superior efficacy and PFS with afatinib versus standard chemotherapy regimens in patients with NSCLC exhibiting *Ex19del* and L858R mutations in *EGFR*, with superior OS also observed in the *Ex19del* cohorts (52–54). A randomized phase IIb trial of afatinib versus gefitinib also demonstrated superior efficacy of afatinib with regards to overall response rate (ORR) and PFS, however, no significant OS benefit was observed (115, 116). In support of this, a meta-analysis of studies that investigated afatinib, gefitinib or erlotinib in patients with NSCLC showed an increase in PFS in patients treated with afatinib compared with those who were treated with erlotinib or gefitinib (117). However, analyses of tumor tissue from patients with NSCLC or lung adenocarcinoma who had received afatinib identified a T790M mutation in up to 70% of samples (49–51). Furthermore, a higher proportion of cells carrying the T790M allele was found in afatinib-resistant than erlotinib-resistant tumor cells (118). This suggested the T790M mutation was still a key mediator of resistance to afatinib in these tumors. Another second-generation irreversible inhibitor, dacomitinib, is also approved for first-line treatment of patients with metastatic NSCLC with the *EGFR* exon 19 deletion or the L858R substitution mutation (55) based on a study that has shown that first-line dacomitinib is superior over gefitinib in improving PFS in patients with NSCLC with sensitizing *EGFR* mutations (58). Previous studies also demonstrated superiority with regards to ORR and PFS over erlotinib (57, 119). Similar to afatinib, however, a mutation leading to the T790M change in the EGFR protein was present in about 50% of patient serum samples at the time of disease progression (56).

The discovery of a common acquired resistance mechanism to first- and second-generation EGFR TKIs through the T790M resistance mutation prompted the development of a third-generation TKI, osimertinib. This irreversible small-molecule EGFR TKI has been shown to be more active against EGFR carrying an activating/sensitizing mutation and T790M resistance mutation than against the wild-type EGFR (120, 121). Osimertinib is indicated for the first-line treatment of patients with metastatic NSCLC whose tumors have an EGFR exon 19 deletion or L858R mutation, or for those patients with T790M mutation-positive metastatic NSCLC who had progressed during or after first- or second-generation EGFR TKI therapy (60). Osimertinib extended PFS of treatment-naïve patients with NSCLC compared with gefitinib-treated patients (69). In addition, osimertinib in combination with chemotherapy showed a significant advantage in prolonging PFS when compared with chemotherapy alone in patients with T790M mutation-positive NSCLC who had progressed on prior systemic therapy, including EGFR TKIs (66, 68). Interestingly, osimertinib has also shown promising efficacy in *in vitro* and *in vivo* studies of glioblastomas exhibiting EGFRvIII mutations (122–124), and a tolerable safety profile in initial clinical studies (125). Previous attempts to inhibit EGFRvIII and glioblastoma in general with both first- and second-generation TKIs and monoclonal antibodies have been relatively unsuccessful, potentially due to the lack of TKI-sensitizing mutations present in NSCLC, the additional obstacle presented by the blood-brain-barrier, and only partial effectiveness of EGFR-targeting antibodies in blocking EGFRvIII (126–129). However, promising preclinical efficacy coupled with increased permeation of the blood-brain-barrier by osimertinib may improve outcomes in this context (124, 125).

Unfortunately, resistance to osimertinib also develops through further acquired mutations in *EGFR* (61). Analysis of patient plasma samples collected in clinical trials that investigated osimertinib as a first-line (61, 67) and a second-line therapy after failure of other first-line TKIs (67) identified an EGFR C797S mutation, which affects the critical site for osimertinib binding, in 7% and 14% of samples, respectively. The C797S mutation frequently occurs in the same allele as the T790M mutation, thus rendering osimertinib completely inactive (64). However, loss of the T790M mutation led to disease progression during treatment with osimertinib (63). A recent study reported that brigatinib, an ALK and EGFR inhibitor, when used in combination with cetuximab, was effective in patients with EGFR-sensitizing mutation/T790M/cis-C797S-positive NSCLC (72, 130). Also, fourth-generation TKIs that can overcome the C797S mutation-conferred resistance to osimertinib are currently undergoing preclinical and early (phase I) clinical development (30, 131–135).

Mechanisms of resistance to TKIs can also occur through compensatory signaling, which bypasses the requirement for signaling through EGFR by activating the same downstream effectors *via* alternative pathways (114). *MET* gene amplification and overexpression of MET protein occurs in approximately 3% of gefitinib-/erlotinib-resistant tumors (33) and results in compensatory signaling involving the ErbB3-dependent activation of the PI3K/AKT pathway (32, 136, 137). *MET* amplification has also been identified in 15% and 19% of plasma samples of patients treated in the first- and second-line setting with osimertinib, respectively (67). *ERBB2* amplification is detected in around 6% of resistant tumors and can also contribute to the oncogenic signaling when EGFR is inhibited by first-generation TKIs (33). Moreover, amplification of *ERBB2* is detected in 2% and 5% of patients with NSCLC that received first- and second-line osimertinib, respectively, and reduces sensitivity of tumors exhibiting T790M mutations to osimertinib (61, 67, 138). Preclinical studies using NSCLC cell lines have also demonstrated that activation of the JAK2/STAT3 pathway can also substitute for EGFR signaling, while increased levels of insulin growth factor-1 receptor (IGFR-1) and constitutive activation of the IGFR-1 pathway was reported in gefitinib- or erlotinib-resistant lung cancer cell lines and gefitinib-resistant tumors in patients with NSCLC (139).

Intercellular communication *via* exosomes is also emerging as a key mediator of resistance to TKIs in NSCLC by influencing cellular signaling (140). A recent study showed secretion of exosomes containing T790M-mutated EGFR by gefitinib-resistant NSCLC tumor cells could horizontally confer gefitinib resistance to sensitive recipient cells (141). Furthermore, transfer of non-coding RNAs *via* exosomes can also modulate response to TKIs, with miR-7 showing the ability to reverse gefitinib resistance through influencing YAP signaling (142), and miR-214 inducing gefitinib resistance through upregulation of signaling by PTEN and AKT (143). Moreover, circular RNA_102481 has been found to be significantly upregulated in NSCLC tumors resistant to EGFR-TKIs, and that silencing of this circular RNA could inhibit EGFR-mediated proliferation and sensitize cells to apoptosis (144). Knowledge of drug-resistance mediated by exosomes and non-coding RNA cargo may have clinical benefit beyond that of being a potential therapeutic target, in that sequencing of miRNAs within exosomes in patient biopsies could establish predictions of response to targeted therapies (144). There is an emerging role for integrative systems biology in identifying novel drug combinations, which may be able to help address the challenge of TKI resistance due to altered cell signaling. One study utilizing bioinformatic approaches to identify driver mutations in TKI-resistant NSCLC lines following RNA sequencing analysis yielded a novel combination of bosutinib and gefitinib that was able to inhibit proliferation and induce apoptosis in these cells (145). Emerging bioinformatic methods which can be used to identify novel drug combinations in cancer are extensively reviewed elsewhere (146).

Outside of the context of signaling, transformation of NSCLC to a different histologic type can also mediate resistance to TKIs. For example, conversion to a squamous cell histologic type was detected in 19% of tumor biopsies from patients, whose disease progressed on first-line treatment with osimertinib (62). Transformation of NSCLC into small-cell lung cancers and the process of epithelial-to-mesenchymal transition have also been cited as mechanisms of resistance to TKIs (30). Key mechanisms of resistance discussed in this section are summarized in (**Table 1**).

### Monoclonal antibodies targeting EGFR signaling

To date, four anti-EGFR monoclonal antibodies, namely cetuximab (chimeric immunoglobulin [Ig] G1), panitumumab (humanized IgG2), necitumumab and nimotuzumab (both humanized IgG1), have been granted approval for clinical use by regulatory bodies (147). These monoclonal antibodies inhibit EGFR signaling by interacting with the EGFR extracellular domain III, and preventing ligand binding to EGFR, receptor dimerization, and signal transduction, thus leading to the internalization of the receptor-antibody complexes and their destruction (147). The binding of cetuximab to EGFR creates steric hindrance prohibiting EGFR heterodimerization with other ErbB receptor family members (148). However, nimotuzumab, despite blocking the EGF-EGFR interaction, does not prevent the formation of an EGFR active conformation, and EGFR remains capable of conveying the basal ligand-independent signaling (149). In addition to this, key anti-EGFR^+^ tumor effects mediated by monoclonal antibodies such as cetuximab are independent of the inhibition of EGFR signaling and occur through the recruitment of cytotoxic natural killer (NK) cells, key effectors of the innate immune system which eradicate tumor cells through ADCC (23, 150). Efficacy data from key clinical trials for cetuximab, panitumumab, and necitumumab are provided in **Table 1**.

Cetuximab is approved for the treatment of patients with SCCHN and EGFR-expressing, *KRAS* wild-type mCRC (73). First-line treatment with cetuximab in combination with radiation therapy improved OS and locoregional disease control compared to radiotherapy alone in patients with locally or regionally advanced SCCHN (49.0 months versus 29.3 months) (95). The combination of cetuximab and platinum-based chemotherapy with fluorouracil, prolonged OS and PFS in patients with recurrent locoregional or metastatic SCCHN (94). Based on studies that have reported an improvement in OS with regimens containing cetuximab in patients with EGFR-expressing, *KRAS* wild-type mCRC (88, 90, 91), cetuximab is recommended in combination with different chemotherapy regimens as first- or second-line therapy, or as a single agent in patients who have failed or are resistant to certain chemotherapy regimens (73). In a recent study, the combination of cetuximab and the BRAF V600E inhibitor encorafenib prolonged OS in patients with BRAF V600E positive-mCRC compared with the combination of cetuximab and chemotherapy (OS= 9.0 months with combination therapy versus 5.4 months in the control group) (92); the combination of cetuximab and encorafenib was recently approved (September 2021) by the FDA for the treatment of patients, whose disease had progressed on one or (73) two prior regimens (151).

Panitumumab monotherapy increases PFS in patients with mCRC who progress during or following fluoropyrimidine-, oxaliplatin-, and irinotecan-containing chemotherapy regimens (97, 99). Interestingly, recent data from the phase III PARADIGM trial evaluating panitumumab in combination with chemotherapy in patients with mCRC have confirmed the results of previous studies (152, 153), that anti-EGFR antibody therapy in this context demonstrates superior efficacy in patients with left-sided tumors than right-sided tumors (96), perhaps reflecting differences in the genetic and molecular underpinnings of the disease highlighted in several previous studies (154, 155). Treatment with panitumumab, as with cetuximab, however, is ineffective in patients with mCRC carrying mutated *KRAS* or *NRAS* (98, 156). Necitumumab in combination with gemcitabine and cisplatin improves OS and PFS in patients with refractory metastatic squamous NSCLC and it has been approved for the first-line treatment in these patients (100, 101). Nimotuzumab has been approved for treatment of patients with SCCHN, glioma and nasopharyngeal cancer in some countries but it has not been recommended by the EMA and FDA for treatment of patients with glioma due to insufficient efficacy and high rates of adverse events (157). However, in some studies of patients with SCCHN, the combination of nimotuzumab and radiotherapy or chemoradiotherapy prolonged OS (60 month OS= 57% nimotuzumab + chemotherapy; 39% nimotuzumab + radiotherapy; 26% chemotherapy only; 26% radiotherapy only) (158).

Despite anti-EGFR inhibitory antibodies being efficacious in distinct subpopulations of patients with mCRC and SCCHN, inherent and acquired resistance to this class of therapy is also common. In patients with mCRC, activating *KRAS* and *NRAS* mutations are a biomarker of primary resistance to cetuximab and panitumumab and the use of these antibodies is not recommended in this setting (88, 90, 91, 156). Inherent resistance is also seen in patients with CRC whose tumors carry *BRAF* V600E (159), *MAP2K1* (74) or *PIK3CA* (160) mutations, *KRAS* (161), *ERBB2*, *MET* or *FGFR1* amplification (74), biallelic *NF1* loss or aberrations in the non-canonical RAS/RAF pathway (111). The mechanisms for inherent and acquired resistance to anti-EGFR monoclonal antibodies seem to overlap in CRC, as *KRAS*, *NRAS* and *EGFR* ectodomain mutations [the latter have also been detected in patients with SCCHN (76, 77)], and *MET* and *KRAS* amplification have also been detected in circulating tumor DNA in patients with acquired resistance to anti-EGFR antibodies (78–83). A recent study evaluated transcriptomic profiles in tumor biopsy material collected from patients with CRC who had progressed on cetuximab monotherapy and showed that acquired resistance to cetuximab was largely mediated by the remodeling of the stromal compartment resulting in the cetuximab-resistant switch to the fibroblast- and growth factor-rich transcriptomic subtype (111). In addition, cetuximab resistance was associated with infiltration of cytotoxic immune cells and elevated expression of programmed death-ligand 1 (PD-L1) and lymphocyte-activation gene 3 (111).

In addition to the anti-EGFR monoclonal antibodies approved for the clinical use, several other anti-EGFR antibodies inhibiting EGFR signaling are currently undergoing clinical development and have been reviewed elsewhere (147).

Considering that amplification of *MET* and overexpression of the MET protein can compensate for the lack of EGFR signaling (74), it was hypothesized that inhibition of both EGFR and MET may be advantageous in combating acquired resistance to anti-EGFR antibodies. A bispecific antibody LY3164530 specific to both EGFR and MET was investigated in a phase I study in patients with different advanced or metastatic cancers, but was subsequently discontinued due to a high rate of adverse events, which is consistent with EGFR inhibition-related toxicities, and poor efficacy (162). Amivantamab (JNJ-61186372), another bispecific antibody targeting both EGFR and MET, was designed with an intention to treat patients with EGFR exon20ins-mutated NSCLC who currently have limited treatment options (163, 164). In an ongoing phase I/II trial in patients with NSCLC, amivantamab achieved a partial response in 36% of patients (164). These promising results in a population of patients with a poor prognosis has led to FDA breakthrough therapy designation for amivantamab (165). However, patients still experienced a high rate of adverse events, with grade ≥3 toxicities being reported in 36% of patients (164). Another bispecific antibody which is undergoing a phase I clinical evaluation is MCLA-158, an antibody-dependent cell-mediated cytotoxicity (ADCC)-enhanced human IgG1 targeting both EGFR and leucine-rich repeat-containing G-protein coupled receptor 5 (LGR5) (166). MCLA-158 showed antitumor activity against *RAS* mutated and wt CRC patient-derived organoids *in vitro* and induced either tumor regression or stasis in esophageal squamous and gastric adenocarcinoma patient-derived xenograft models expressing LGR5 and EGFR (166). In patients with mCRC, who progressed after receiving oxaliplatin, irinotecan and fluoropyrimidines, and EGFR monoclonal antibodies, MCLA-158 was well tolerated and no dose limiting toxicity was achieved (166). However, despite these promising data, there is a risk that therapies co-targeting EGFR and another cell-surface kinase receptor may experience similar issues with toxicity, due to the critical role of EGFR activity in healthy tissues (167).

### On-target off-tumor toxicity of EGFR signaling-inhibiting therapeutic agents

One major concern relating to both EGFR TKIs and inhibitory anti-EGFR monoclonal antibodies is the high rate of adverse events and the frequently occurring cutaneous toxicities (167). EGFR plays a critical role in maintaining homeostasis of healthy mesenchymal, epithelial, and neurologic tissues, and the inhibition of the basal EGFR signaling can cause cell death, impaired cell proliferation and abnormal cell differentiation in these healthy tissues (167–169). Dermatologic toxicities occur in around 45–100% of patients treated with EGFR TKIs and monoclonal antibodies against EGFR (167), and the underlying cause for these toxicities is linked to a non-redundant role of EGFR in regulating normal keratinocyte growth, survival, differentiation, and migration, and maintenance of an adequate immune response in the skin (167, 169). Epithelia in normal tissues respond to injury by promoting proliferation of epithelial cells, and the inhibition of EGFR signaling interferes with the regeneration of epithelial surfaces, such as skin (168) and the gastrointestinal lining (169, 170). Persistent tissue damage destroys epithelial barriers, leading to pathogen invasion and acute inflammation, which perpetuates an even larger influx of immune cells leading to further tissue injury (171). As the barrier cannot be closed, the physiological balance between inflammation and tissue regeneration is disturbed.

A meta-analysis of toxicity data pooled from 28 randomized controlled trials, which investigated EGFR TKIs in patients with various cancers, reported diarrhea, rash, mucositis, alanine aminotransferase increased, and skin reaction as most common any grade adverse events, while the most frequently reported grade ≥3 toxicities included mucositis, pain, metabolism and nutrition disorders, diarrhea, dyspnea, and hypertension (172). Statistically significant differences in the risk ratios emerged for the different generation lines of TKIs (172). Second-generation TKIs (afatinib, dacomitinib, lapatinib, neratinib, and vandetanib) were associated with the highest risk of high-grade diarrhea compared with first- (gefitinib and erlotinib) or third-generation (osimertinib) TKIs and were more likely to cause any grade fatigue and nausea, and high-grade vascular disorders and fatigue than first-generation TKIs (172). Furthermore, in comparison to treatment with first-generation EGFR-TKIs (gefitinib or erlotinib), osimertinib had a higher rate of cardiac toxicity, manifesting in QT prolongation in 10% of patients who had received osimertinib compared with 4% of patients treated with EGFR-TKIs, and cardiac failure in 4% of patients treated with osimertinib and 2% with EGFR-TKIs (69). Effects of TKIs on hair growth include hypertrichosis, trichomegaly and a range of scalp hair changes (173).

Approximately 10–20% of patients treated with anti-EGFR antibodies experience grade 3/4 toxicities (73, 99, 174), including acneiform rash, radiation dermatitis enhancement, pruritus, mucositis, xerosis/fissures, paronychia, and gastrointestinal toxicity, all of which may lead to greatly reduced patient quality of life and antibody dose reduction, interruption or complete cessation of the treatment (73, 99, 174). There is also a risk of secondary infections occurring in these patients, which can be fatal (73, 99), and up to 96% of patients can also experience significant gastrointestinal disorders (73, 99). In clinical trials investigating cetuximab, cardiopulmonary arrest was reported in 2–3% of patients (73).

Taken together, these data demonstrate the critical role of EGFR kinase activity in normal tissue homeostasis and the response to injury, and the limitations of the use of TKIs and EGFR-targeting antibodies in patients with cancer due to their detrimental effect on normal tissue physiology. The necessity of management of dermatologic and gastrointestinal toxicities may require the EGFR-targeted treatment to be temporarily interrupted to allow patients to recover, and this may reduce the effectiveness of the therapy and potentially lead to acceleration of disease progression (167, 174).

## Engaging adaptive immunity in targeting EGFR-expressing tumors

The need for novel therapeutic approaches has emerged to circumvent shortcomings related to acquired resistance and on-target off-tumor toxicities induced by EGFR TKIs and anti-EGFR antibodies. One of these approaches is the development of therapeutic agents that exploit cell-surface EGFR as a decoy to direct the activity of key components of the adaptive immune system such as CD4+ T cells, B lymphocytes, and the cytotoxic CD8+ and γδ/αβ T-cell receptor positive (TCR+) T cells to EGFR-expressing cancer cells and to destroy them in a target-specific manner.

### Immune checkpoint inhibitors

One such therapeutic strategy involves blocking inhibitory immune checkpoints using immune checkpoint inhibitors (CPIs). Several different molecules expressed on immune cells and cancer cells convey inhibitory and stimulatory signals, called immune checkpoints, to regulate cancer immunity. Cytotoxic T lymphocyte-associated protein-4 prevents activation of T lymphocytes, while programmed cell death protein-1 (PD-1) upon binding to its ligand PD-L1 inactivates the ability of cytotoxic T lymphocytes to destroy tumor cells (175). CPIs target these molecules and have revolutionized the treatment of many patients with different cancers (175). Two CPIs, nivolumab and pembrolizumab have been approved for the treatment of SCCHN following pivotal phase III trials. In the Checkmate-141 phase III trial, nivolumab demonstrated superior OS over standard-of-care therapy in patients with relapsed SCCHN (7.5 months vs. 5.1 months) (176). Similarly, in the Keynote-040 trial, patients treated with pembrolizumab monotherapy demonstrated superior OS over those treated with chemotherapy and cetuximab (8.4 months vs. 6.9 months) (177). This was observed to an even greater extent in the Keynote-048 trial in patients treated with pembrolizumab in combination with chemotherapy, versus chemotherapy plus cetuximab (13.0 months vs. 10.7 months) (178). Pembrolizumab with chemotherapy has also shown efficacy in mCRC, improving PFS over chemotherapy, however, no significant improvement in OS was observed (179).

In the context of NSCLC, PD-1 and PD-L1 inhibitors such as pembrolizumab, nivolumab, and atezolizumab in combination with chemotherapy have become the standard-of-care in frontline therapy after demonstrating significant improvements over chemotherapy with regards to ORR, PFS, and OS in patients without EGFR-activating mutations (180). However, much lower efficacy has been noted if EGFR-driver mutations are present (181). As EGFR signaling can induce a tumor-suppressive microenvironment through upregulation of factors such as IL-6, TGF-β and progranulin, and induces PD-L1 expression, and PD-L1 can mediate resistance to TKIs through upregulation of YAP1, there is a clear preclinical rationale for the use of combination therapies simultaneously targeting PD-L1 and EGFR signaling (181). Trials investigating combinations of TKIs with CPIs, however, have raised safety concerns, with one study of osimertinib in combination with durvalumab leading to a 38% incidence of pneumonitis, compared with 2.9% and 2% incidence of pneumonitis with osimertinib or durvalumab monotherapy, respectively (182). A high (39%) incidence of Grade 3 and 4 adverse events was also observed in patients receiving erlotinib and atezolizumab (183).

There is also a rationale for the use of CPIs in combination with EGFR-targeting antibodies such as cetuximab to treat other tumor types such as SCCHN and mCRC, with a predicted synergistic effect due to the ability of PD-L1 inhibitors to alleviate immunosuppression in the tumor microenvironment, and the ability of cetuximab to stimulate cells of the innate and adaptive immune system to induce anti-tumor ADCC (150). A pilot study of cetuximab plus radiotherapy and avelumab in patients with advanced SCCHN unfit for cisplatin treatment demonstrated manageable toxicity and transient immune-related toxicity, setting the scene for larger trials in this setting (184). Additionally, correlative science data from a phase I/II trial of cetuximab in combination with pembrolizumab demonstrated an increase in intratumoral CD3^+^ CD8^+^ cytotoxic T cells, a decrease in cytotoxic T cells in the peripheral blood, and decreased levels of PD1^+^ cytotoxic T cells in both the tumor and peripheral blood, consistent with therapy-related changes in the tumor microenvironment (185). Further combination regimens involving anti-EGFR antibody cetuximab and CPIs are currently being investigated in a number of phase I−III clinical trials in patients with EGFR-expressing tumors (150).

### EGFR-targeted CAR-T cell therapies

CAR-T cell therapies are T cells that have been modified *ex vivo* to target a specific tumor cell-surface antigen and thereby to use the adaptive immune system to destroy cancer cells. The first-generation CAR-T cells are engineered to express receptors comprising an extracellular single-chain variable fragment (scFv), which recognizes a specific tumor cell-surface antigen, a transmembrane domain, and an intracellular part containing immunoreceptor tyrosine-based activation motifs and a co-stimulatory domain, that is crucial for the T-cell activation, proliferation, persistence, and cytotoxicity (186). Two or more co-stimulatory domains are typically incorporated in the second- and third-generation CAR-T cell therapies (186).

In general, CAR-T therapies have been reported to be efficacious in small populations of patients with specific cancers, mainly those with hematologic malignancies, but in patients with solid tumors, their effectiveness has been limited (187, 188). The advantage of using therapeutic agents that engage the adaptive immune response is that the response is targeted to cells expressing specific antigens. However, the tumor microenvironment is usually not conducive to therapy, with poorly vascularized and hypoxic tumor regions preventing CAR-T cell homing to tumors and the anti-inflammatory tumor microenvironment being detrimental to the CAR-T cell survival (29). In addition, tumors often show heterogeneous expression of target antigens, and the lack of universally expressed cancer antigens significantly reduces the antitumor activity (186).

CAR-T therapy targeting EGFR is in early clinical development for numerous types of cancer (186, 189, 190). Several studies ongoing in patients with glioblastoma are investigating CAR-T cell therapies targeting the EGFRvIII mutant, which is identified in around 31% of glioblastomas (191), but not in healthy tissues, therefore, reducing the risk of off-target effects and toxicity (186) (**Table 2**). A phase I trial in patients with glioblastoma found that CAR-T cells targeting EGFRvIII specifically accumulated in tumors and showed a good safety profile (29). No EGFR-associated toxicities, such as rash and diarrhea, were reported, but clinically significant neurologic events occurred in three of 10 patients (29). Most patients showed a complete loss or reduced expression of EGFRvIII in their tumors, but the tumors also expressed anti-inflammatory markers and secreted cytokines, which reduced the effectiveness of the CAR-T-EGFRvIII cell therapy (29). Moreover, tumors showed intratumoral heterogeneity of EGFRvIII expression levels, suggesting that the lack of uniformity in target expression in tumor tissue may contribute to the suboptimal efficacy (13). In support of this, a pilot dose-escalation phase I trial investigated patients with EGFRvIII-expressing recurrent glioblastoma who were treated with a third-generation CAR-T-EGFRvIII cells and reported no clinically meaningful effect in these patients (194). Another phase I study investigated CAR-T therapy targeting EGFR in patients with EGFR-expressing biliary tract tumors (195). Ten of 17 treated patients had stable disease and 1 of 17 patients showed a complete response (195). CAR-T-EGFR cell therapy was well tolerated in this setting (195) (**Table 2**). Moreover, the enrichment of central memory T cells in the infused CAR-T-EGFR cells showed a good correlation with the persistence of CAR-T-EGFR cells in patients (195). In another phase I study, EGFR-targeted CAR-T cell infusions were well tolerated without severe toxicity in patients with NSCLC, and of 11 patients, two had partial response and five had stable disease for up to 8 months (196) (**Table 2**). CAR-T-EGFR cell therapy has also been shown to be well tolerated in patients with metastatic pancreatic cancer in a phase I trial, where out of 14 evaluable patients, four achieved partial response and eight had stable disease for 2–4 months (193) (**Table 2**).

**Table 2:** Safety and clinical response to CAR-T therapies in phase I clinical trials.

Trial Identifier Number	Patients (N)	CAR-T Cell Therapy	Diagnosis	Grade ≥3 AEs in ≥10% of patients, n (%)		Clinical Response	Reference
NCT03182816	9	CAR-T-EGFR	EGFR+ NSCLC	Grade 1 to 3 fever	7 (78)	Median PFS: 7.13 months (range 2.71–17.10 months) Median OS: 5.63 months (range 8.82–22.03 months) PR: 1/9 (11.1%) SD: 6/9 (66.7%)	Zhang Y, et al., 2021 (192)
NCT01869166	16	CAR-T-EGFR	EGFR+ metastatic pancreatic carcinoma	Lymphocytopenia Dermatitis herpetiformis Pleural effusion Pulmonary interstitial exudation	6 (38) 2 (13) 2 (13) 2 (13)	Median OS: 4.9 months (range 2.9–30 months) Median PFS: 3 months (range 2–4 months) ORR: 4/14 (29%) DCR: 12/14 (86%) PR: 4/14 (29%) SD: 8/14 (57%)	Liu Y, et al., 2020 (193)
NCT01454596	18	CAR-T-EGFRvIII	Recurrent EGFRvIII+ glioblastoma	Lymphopenia^a^ Neutropenia^a^ Thrombocytopenia^a^ Anemia^a^ Bacteremia^b^ Dyspnea/hypoxia^c^ Hypotension^d^ Febrile neutropenia^e^ Transaminitis	18 (100) 18 (100) 18 (100) 9 (50) 8 (44) 2 (11) 2 (11) 2 (11) 2 (11)	Median OS: 6.9 months (IQR 2.8–10) Median PFS: 1.3 months (IQR 1.1–1.9)	Goff SL, et al., 2019 (194)
NCT02209376	10	CAR-T-EGFRvIII	EGFRvIII+ glioblastoma	Edema cerebral Seizure	2 (20) 2 (20)	Median OS: 251 days (~8 months) PFS: NE	O’Rourke DM, et al., 2017 (29)
NCT01869166	19	CAR-T-EGFR	EGFR+ cholangiocarcinoma (N=14) EGFR+ gallbladder carcinoma (N=5)	Lymphopenia Acute fever/chill	16 (84) 3 (16)	Median PFS: 4 months (range 2.5–22 months) CR: 1/17 (6%) SD: 10/17 (59%)	Guo Y, et al., 2018 (195)
NCT01869166	11	CAR-T-EGFR	EGFR+ advanced R/R NSCLC	NR		PR: 2/11 (18%) SD: 5/11 (45%)	Feng et al., 2016 (196)

a Expected to be due to lymphodepleting chemotherapy.

b Asymptomatic.

c Includes 1 treatment-related mortality (Grade 5).

d Not associated with sepsis.

e Without bacteremia.

CAR-T, chimeric antigen receptor T-cell therapy; CR, complete response; DCR, disease control rate; EGFRvIII, epidermal growth factor receptor variant III; IQR, interquartile range; N, number; NE, not evaluable; NR, not reported; NSCLC, non-small cell lung cancer; ORR, objective response rate; OS, overall survival; PR, partial response; R/R, relapsed/refractory; SD, stable disease.

However, other studies have shown CAR-T therapy to increase the risk of severe adverse events. Neurologic toxicities have been reported with CAR-T therapy (197, 198), with symptoms including encephalopathy, headache, tremor, aphasia and focal weakness (198). In one study, 20% of neurotoxicity events were of grade ≥3 severity (198). However, the most common toxicity experienced by patients is cytokine release syndrome (CRS) (197), which is a systemic inflammatory response to cytokines, that can lead to organ damage, and death and occurs when effector immune cells cross-react, triggering target-independent cytokine release. Therefore, patients receiving CAR-T therapy must be monitored for CRS, as treatment needs to be prompt and aggressive (197).

One novel EGFR-targeting CAR-T therapy for overcoming CAR-T-associated toxicity is currently in early preclinical development. The UniCAR system uses two separate modules; the first is the UniCAR-T cells that are inert, and the second is composed of a target module, containing an antigen binding domain linked to the E5B9 peptide epitope (199). The UniCAR T cells become activated only when the crosslinking to the target module *via* its E5B9 peptide epitope takes place (199). This system shows promise in cell models and can target the UniCAR-T cells effectively to tumor cells and only becomes active in the presence of a tumor antigen (199). Encouragingly, the UniCAR-T-EGFR cells also show activity against cancer cells expressing low levels of EGFR (199). However, the efficacy of this system in a clinical setting is currently unknown.

### Bispecific antibodies

Another therapeutic modality that allows the coupling of specific tumor antigens with the immune response cells, namely T or NK cells, is bispecific antibodies. These antibodies bind concomitantly to two different antigens, one expressed on cancer cells and another on immune cells (191, 200). One bispecific antibody in preclinical development targets EGFR-expressing tumors by using an anti-EGFR IgG portion of the molecule, and engages the PD-1 on T cells *via* the scFv of an anti-PD-1 antibody (200). This antibody simultaneously inhibits EGFR signaling, activates T cells and initiates a tumor immune response by blocking the interaction between PD-1 on T cells and PD-L1 on tumor cells, and also induces strong ADCC (200). When tested in cellular cytotoxicity assays *in vitro*, the antibody induced EGFR-dependent cell death, and in tumor-bearing animals, recruited T cells to tumor xenografts (200).

Another bispecific antibody (hEGFRvIII-CD3) comprising two scFv fragments (one specific to the EGFRvIII antigen and another to the CD3 epitope) was designed to create a bridge between EGFR-expressing cancer cells and CD3+ T cells, and to prevent a non-specific targeting to EGFR-negative cells. The hEGFRvIII-CD3 antibody activated CD3+ T cells in a target-specific manner, induced the release of proinflammatory cytokines, prompted T-cell proliferation, and caused significant lysis of malignant glioma cell lines and patient-derived EGFRvIII-expressing malignant glioma samples *in vitro* (201). The hEGFRvIII-CD3 antibody also showed antitumor activity in several preclinical malignant glioma models, and significantly extended survival of experimental animals (201).

A novel bispecific T-cell engager AMG 596, which comprises two single-chain variable fragments with one being specific for EGFRvIII and another for CD3, was recently investigated in glioblastoma preclinical models (202). The simultaneous engagement of EGFRvIII expressed on glioblastoma cells and CD3 on T cells led to a potent antitumor activity against the EGFRvIII-expressing glioblastoma cells *in vitro*, while the treatment of mice bearing EGFRvIII-expressing orthotopic tumors significantly extended OS of experimental animals (202). Moreover, the treatment of cynomolgus monkeys with AMG 596 showed a good safety profile (202). A new type of a bispecific antibody that engages simultaneously EGFR on tumor cells and CD3 on T cells was designed to prevent its binding to EGFR expressed in healthy tissues (a masked form) but to become activated by proteases (an unmasked form) in the tumor microenvironment. Proteolytically activated, but not inactive, EGFR-CD3 T cell-engaging antibody showed specific EGFR-dependent tumor cell killing *in vitro* and caused tumor regression in preclinical tumor models, while a nonhuman primate study established that the maximum tolerated dose increased by 60-fold when the EGFR-CD3 antibody was administered in the masked form. Therefore, a localized activation of a bispecific antibody is likely to reduce on-target toxicity and increase its therapeutic index (203).

Although the preclinical investigation of these bispecific antibodies is showing promising results, they have not yet entered clinical development.

## Therapeutic agents exploiting the innate immune system to target EGFR-expressing cancer

To overcome T-cell-associated toxicity issues, a novel approach is being developed that deploys immune cells mediating the innate immune responses to target specific cell-surface epitopes on cancer cells (204).

**Figure 2:**
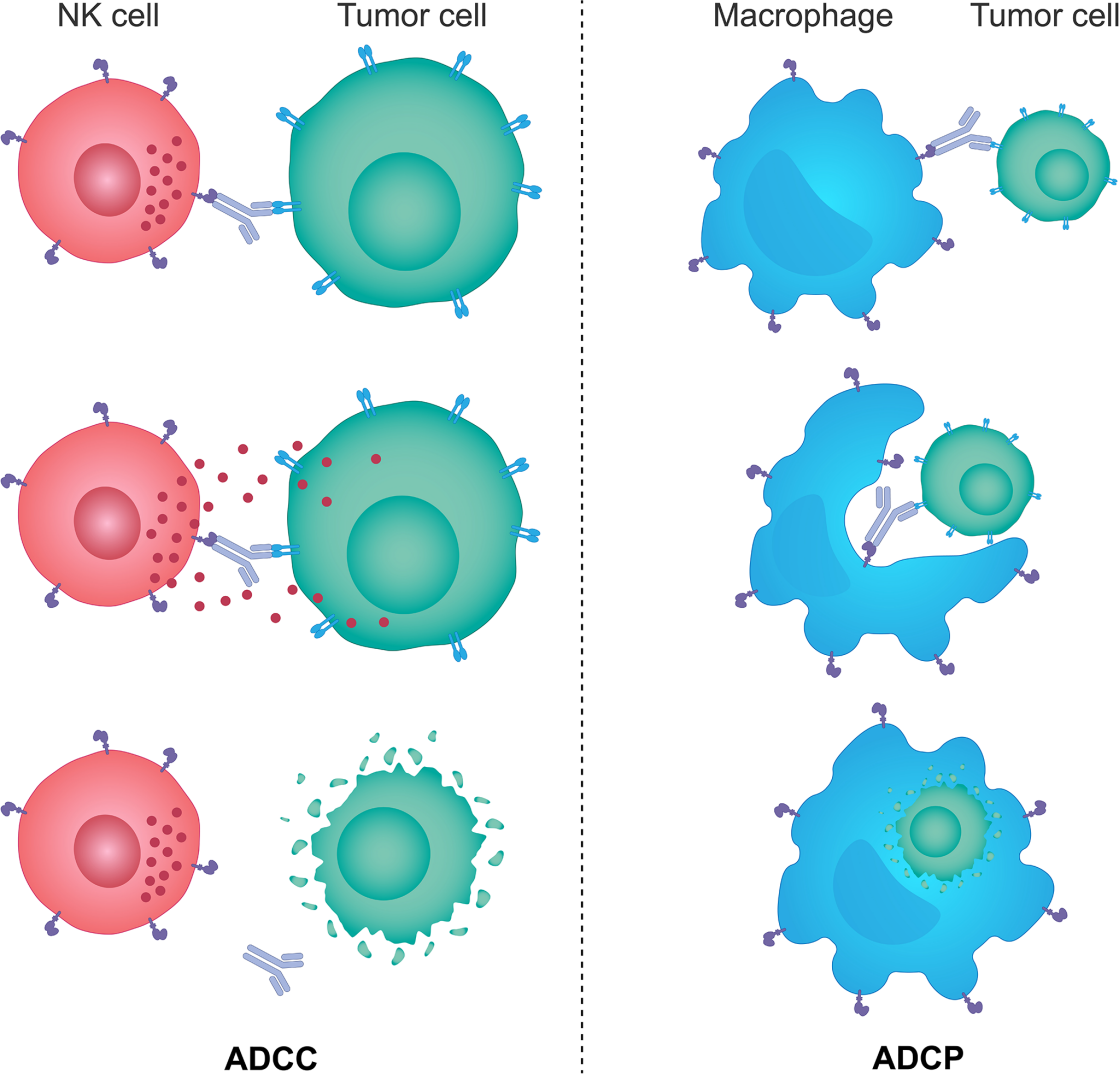
The mechanisms of ADCC and ADCP response. Monoclonal therapeutic antibodies designed to target specific tumor cell antigens can also use their Fc portion of the immunoglobulin to anchor NK cells and macrophages through specific Fc receptors expressed on the surface of these cells. Such interactions trigger activating signals downstream of Fc receptors in NK cells and macrophages and lead to NK cell-mediated ADCC and macrophage-mediated ADCP responses. NK cells brought in the vicinity of target tumor cells by monoclonal antibodies kill those cells predominantly through the perforin/granzyme cell death pathway, while activated macrophages engulf antibody-opsonized target tumor cells and degrade them through acidification of the phagosome. ADCC, antibody-dependent cellular cytotoxicity; ADCP, antibody-dependent cellular phagocytosis; Fc, fragment crystallizable; NK, natural killer.

The innate immune system provides the immediate response to infection and foreign antigens and plays a key role in tumor immunosurveillance through recognition and destruction of transformed cells both prior to and following the establishment of a tumor (205–207). Innate immunity is also essential for the onset and maintenance of the adaptive immunity; by stimulating innate immunity it is possible to harness both sides of the immune system (204). The innate immune system comprises innate lymphoid cells, including a specialized population of NK cells, macrophages, neutrophils, dendritic cells, mast cells, basophils, eosinophils and γδ T cells (205). The key cells involved in tumor immunity are the NK cells, which induce ADCC, and scavenging macrophages, which phagocytose tumor cells *via* the antibody-dependent cellular phagocytosis (ADCP) mechanism (**Figure 2**); both can also stimulate the downstream activation of the adaptive immune response (205, 206, 208, 209). High levels of tumor-infiltrating NK cells have been shown to be associated with a favorable prognosis in numerous solid tumors, and several studies have also shown that intact cytolytic function of NK cells is important in protecting from the development of some types of malignancies (210–213). However, the more advanced tumors can upregulate the expression of inhibitory molecules inactivating NK cells, and escape from immunosurveillance (213–216).

### EGFR-targeting antibodies and ADCC

ADCC is a process by which cells expressing specific antigens are recognized by antibodies, which also interact *via* a constant region (Fc) with the Fc-gamma receptors (FcγR) on the surface of immune effector cells, leading to the direct lysis of target cells (217). NK cells are considered to be the key effectors of the innate immune system mediating ADCC due to their unique expression of activating FcγRs, such as FcγR IIIa (CD16a) and FcγR Iic (CD32a) (217).

As previously alluded to, some EGFR-targeting antibodies, approved for the use in patients with mCRC and SCCHN, not only inhibit ligand-induced EGFR activity and signaling, but also elicit ADCC through the interaction between their IgG1 Fc region and the FcγR-expressing effector cells, typically NK cells, leading to the destruction of EGFR-expressing cancer cells (148, 217). Cetuximab has been shown to be capable of inducing NK cell mediated ADCC in preclinical, clinical, and *ex vivo* assays, which are extensively reviewed elsewhere (150). This is not the case for panitumumab, which contains an IgG2 Fc region and stimulates ADCC to a much lesser extent (23). This has been suggested to account for differences in the clinical efficacy of cetuximab and panitumumab in patients receiving either cetuximab or panitumumab in combination with chemotherapy regimens in SCCHN, where cetuximab has been shown to increase OS, but panitumumab does not (94, 95, 150, 218, 219). This again suggests that levels of ADCC and intratumoral immune activity play a key role in patient responses in these tumors. Interestingly, a study has shown that the FcγR genotype in patients with *KRAS* wild-type mCRC may correlate with the ADCC-mediated responsiveness to cetuximab, thus potentially providing a rationale for patient stratification (220). FcγRIIa H/H and H/R alleles of the FcγRIIa-H131R polymorphism elicited significantly higher ADCC compared with the R/R alleles, and the FcγRIIIa V/V and V/F alleles of the FcγRIIIa-V158F polymorphism induced stronger ADCC than the F/F alleles (220). Moreover, patients with the FcγRIIIa 158V allele had significantly longer PFS than those with the 158F/F allele (220). Another study showed that the combination of cetuximab and interleukin 12 in patients with unresectable primary or recurrent SCCHN resulted in higher ADCC and prolonged PFS (221). Similar to cetuximab, nimotuzumab, an IgG1 isotype antibody, has been shown to be capable of exerting a detrimental effect on EGFR-expressing cancer cells by NK cell-mediated ADCC in patients with SCCHN. Activation of NK cells led to dendritic cell maturation and priming of EGFR-specific CD8+ T cells (222).

Other EGFR-targeting antibodies, being tested in early stages of clinical development, have demonstrated superior ADCC responses when compared with cetuximab (163, 223). Imgatuzumab, a monoclonal antibody inhibiting EGFR signaling, induced a more robust ADCC response than cetuximab (223). However, in patients with *KRASexon2*-WT and *KRASexon2*-mutant-CRC, despite the ability to initiate a stronger ADCC response, the combination of imgatuzumab and FOLFIRI did not lead to improved PFS when compared with chemotherapy alone or cetuximab treatment (median PFS *KRASe2*-WT = 7.3 months with imgatuzumab + FOLFIRI, 6.1 months with cetuximab + FOLFIRI; median PFS *KRASe2*-mutant = 5.2 months imgatuzumab + FOLFIRI, 4.3 months with FOLFIRI only) (224, 225). The EGFR-MET targeting bispecific antibody amivantamab has also been shown to induce more robust ADCC than cetuximab, and a direct correlation was established between the ADCC activity and secreted interferon γ levels in preclinical NSCLC models with *EGFR* exon 20 insertions (163). Amivantamab has since been granted accelerated approval by the FDA for patients with NSCLC exhibiting EGFR exon 20 insertions who have progressed on platinum-based therapies, where its high-capacity for ADCC induction may be contributing to its favorable efficacy (226).

The capability for ADCC induction by anti-EGFR targeting antibodies may also be enhanced through combination with recently developed covalent inhibitors of KRAS G12C, AMG510 and MRTX849, in patients with tumors that harbor this mutation. Recent *in vivo* data has suggested that the inhibition of KRAS G12C using AMG510 creates a pro-inflammatory tumor microenvironment, promoting the anti-tumor activity of immune cells alone and in combination with immune CPIs (227). As the onset of the pro-inflammatory tumor microenvironment has been shown to be synonymous with the increased infiltration of immune cells such as T lymphocytes into the tissue, CD16^+^ subsets of which can mediate ADCC (228), with both AMG510 (227) and MRTX849 (229), combining KRAS G12C inhibitors with anti-EGFR antibodies, which stimulate ADCC, may have synergistic effects in these tumors. An ongoing phase III trial is currently investigating the impact on survival of MRTX849 in combination with cetuximab versus chemotherapy in patients with advanced CRC harboring *KRAS G12C* mutations (NCT04793958) (230).

### CAR-NK-EGFR cells

NK cells, can be primed and modified *ex vivo*, in a similar manner to CAR-T cells (231), to express a CD38-CD3ξ domain required for NK cell signaling and scFv antibody fragments to introduce specificity to a range of diverse tumor antigens, including those targeting the cell-surface EGFR or/and EGFRvIII. The therapeutic use of CAR-NK cells may have significant advantages over the CAR-T cell therapy, as CAR-NK cells have a better safety profile due to a low potential to induce CRS, neurotoxicity and graft-versus-host disease, and the ability to exert the CAR-independent cytotoxicity (232).

Different CAR-NK-EGFR cells showed target-specific cytotoxicity in *in vitro* cell-based assays, reduced xenograft tumor growth in renal cell carcinoma and triple-negative breast cancer animal models, and significantly extended survival of intracranial tumor-bearing animals in metastatic breast cancer and glioblastoma models (231, 233–236). However, despite promising results in the preclinical setting, one study found that CAR-NK cell treatment of animals with glioblastoma failed to inhibit tumor progression and led to a pseudo-progression phenotype (237). In general, CAR-NK cell-based therapies face several challenges due to the short life-span of CAR-NK cells in the absence of cytokines, the need for expansion and activation *ex vivo*, inactivation by tumor cells and the tumor microenvironment (28), and cross-reactivity leading to NK cell fratricide (209). A number of clinical trials have been initiated to test CAR-NK cells, with specificity to antigens other than EGFR, in patients with hematologic malignancies and solid tumors (238). In heavily pretreated patients with relapsed or refractory B-cell hematologic malignancies, CAR-NK-CD19 therapy was found to be safe, and no CRS, immune effector cell-associated neurotoxicity syndrome or graft-versus-host disease were reported (239), suggesting that NK-CAR cells may have a superior toxicity profile compared with that shown by CAR-T cell therapies. However, although a promising method for targeting EGFR, these therapies are in an early stage of development and further work is needed to establish CAR-NK cells as effective therapies in solid tumors (28).

### An innate cell engager as a novel EGFR-targeting modality

A novel therapeutic modality, the innate cell engager, has been developed to bind simultaneously to NK cells or macrophages *via* a distinct epitope on CD16A recognized by the CD16A-specific antibody variable domains, and to cancer cells *via* variable antibody domains specific to cancer epitopes in order to potentiate the NK cell- or macrophage-dependent destruction of cancer cells in solid tumors (209). This was hypothesized to prevent tumor escape from immunosurveillance, which depends on the balance between activating and inhibitory NK cell populations, due to increased cancer cell killing (209). Innate cell engagers can be engineered as bispecific or multispecific molecules and are derived from the fit-for-purpose redirected optimized cell killing antibody platform, comprising an array of bispecific and multispecific antibodies (209). These antibodies bind CD16A independently of the CD16A allotype, do not cross-react with the Fc binding site, thereby avoiding competition with the body’s own circulating serum IgG, do not exhibit NK cell fratricide, and bind to specific tumor cell antigens even when expressed at low levels. Consequently, innate cell engagers link tumor antigens, such as EGFR, to FcγRIIIa (CD16A) on NK cells or macrophages and activate ADCC and ADCP, resulting in tumor cell killing and phagocytosis of tumor cells, respectively (209), and potentially the reduction of a tumor mass.

**Figure 3:**
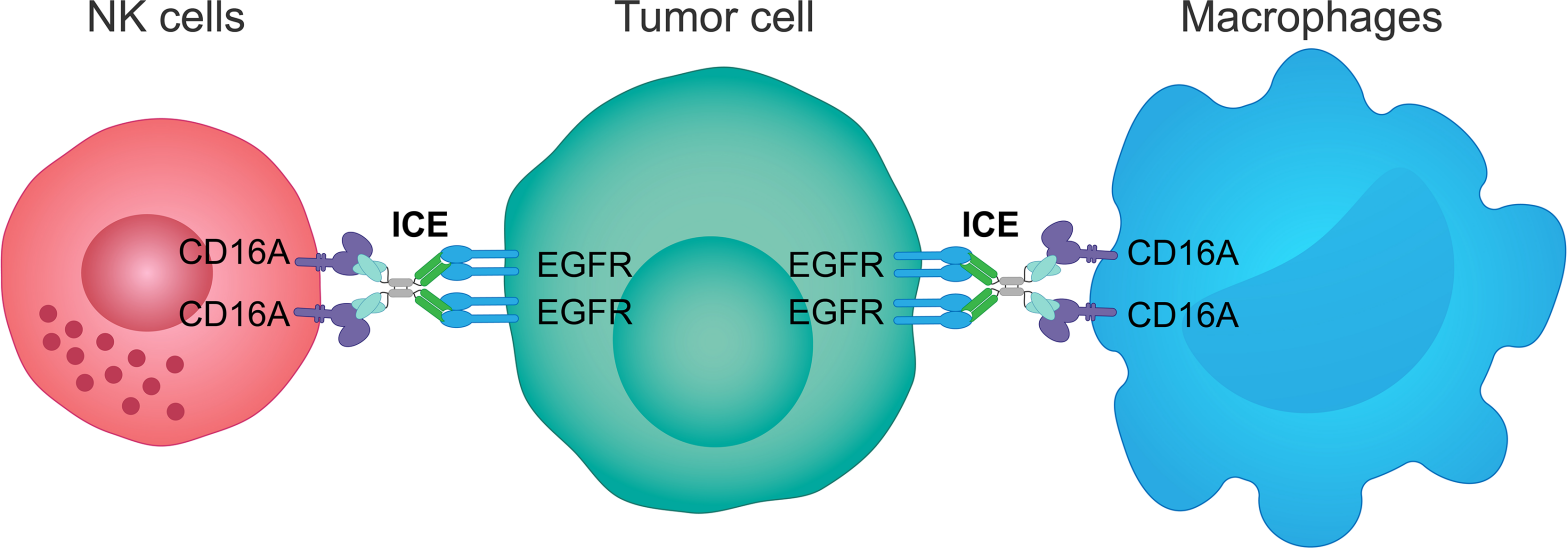
Mechanism of action of an innate cell engager targeting EGFR and CD16A. AFM24, a fully human tetravalent bispecific innate cell engager, binds simultaneously the CD16A receptor on NK cells or macrophages, with a much higher affinity than monoclonal antibodies, and the EGFR antigen on the surface of tumor cells. This creates a bridge between innate immune cells and EGFR-expressing tumor cells enabling ADCC mediated by NK cells and ADCP mediated by macrophages. ADCC, antibody-dependent cellular cytotoxicity; ADCP, antibody-dependent cellular phagocytosis; EGFR, epidermal growth factor receptor; ICE, innate cell engager; NK, natural killer.

**Figure 4:**
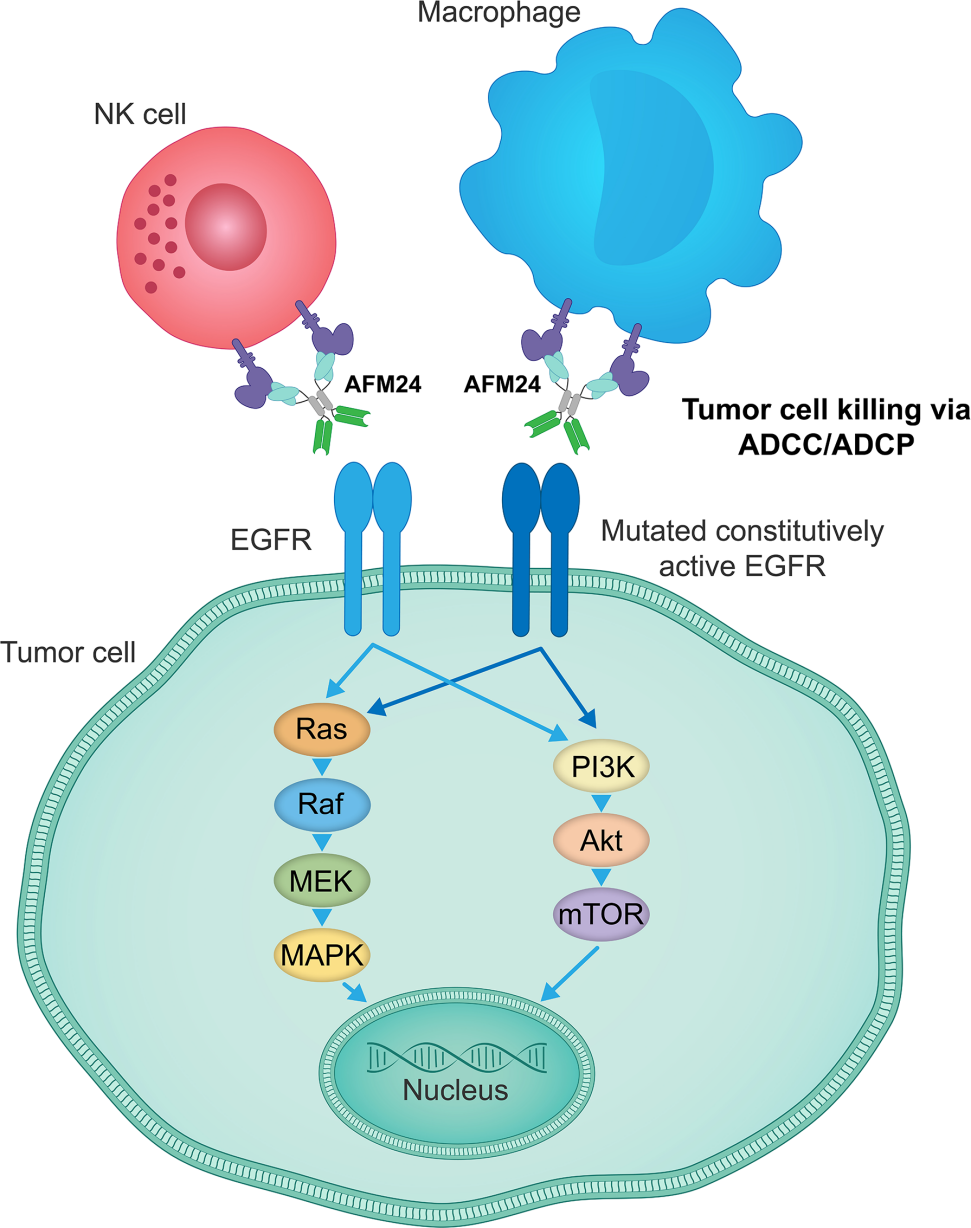
AFM24 activity is independent of EGFR signaling function. AFM24-mediated killing of EGFR-expressing tumor cells, by inducing ADCC and ADCP responses, does not rely on the EGFR activity, its mutational status or the disruption of downstream signaling pathways. ADCC, antibody-dependent cellular cytotoxicity; ADCP, antibody-dependent cellular phagocytosis; EGFR, epidermal growth factor receptor.

AFM24 is a tetravalent bispecific innate cell engager that binds simultaneously to CD16A on NK cells and macrophages and EGFR that is expressed on the tumor cell surface (**Figure 3**). It has been designed to prevent cross-linking of effector cells, which is expected to reduce the risk of target-independent activation, cytokine release and subsequent CRS (209, 240, 241). AFM24 shows high specificity for CD16A and robust binding to NK cells and macrophages in *in vitro* assays (209, 240, 241). Preclinical studies have found that AFM24 binds EGFR-expressing tumors with high affinity and induces targeted, dose-dependent and potent lysis by NK cells and phagocytosis by macrophages (241). This was independent of the *KRAS/BRAF* mutation status and EGFR expression levels of tumor cells, thus suggesting that resistance mechanisms observed with the therapeutic agents targeting EGFR activity and signaling may not be relevant in this setting (**Figure 4**) (241).

Preclinical data generated in cynomolgus monkeys have shown no off-target side effects, no evidence of CRS, and no dermatologic toxicities characteristic to EGFR signaling inhibitors (TKIs and anti-EGFR antibodies) (241). The observed improved toxicity profile in animal models may be determined by the inherent feature of the AFM24 design associated with a minimal effect on the EGFR signaling pathway. The tumor-associated microenvironment often shows persistent inflammation induced and maintained by a continuous production of different pro-inflammatory molecules, tumor vascularization, and infiltration of immune cells mediating the innate and adaptive immune responses (240). Considering that NK cells and macrophages are extremely abundant in tumor tissues, AFM24 can effectively utilize these cells to cause extensive tumor damage and to further promote inflammation and the antitumor immune response. Experiments using AFM24 at up to 75 mg/kg dose once weekly for 28 days in cynomolgus monkeys suggest that healthy tissues, including skin, can be spared as any tissue damage will be easily repaired by EGFR signaling due to its intact catalytic activity (240).

AFM24 has shown activity in several tumor cell lines of different origin, which suggests that it may be beneficial in patients with a range of solid cancers (240). This is reflected in the study design for the ongoing phase I/IIa trial (NCT04259450), which is investigating AFM24 in patients with advanced or metastatic EGFR-expressing solid cancers, including but not limited to colorectal, lung, gastric, esophageal, pancreatic, head and neck, breast, ovarian, cervical, urothelial, and renal cancer (242).

### The potential and advantages of EGFR-specific innate cell engager combinations with other treatment modalities

Conventional EGFR-targeting therapies that inhibit EGFR kinase activity and signaling have a detrimental effect on healthy tissues, particularly the skin and the gastrointestinal epithelial lining, where EGFR plays a crucial role in maintaining tissue homeostasis. On-target off-tumor effects limit the effectiveness of therapeutic interventions targeting EGFR signaling and combinations with therapeutic agents, which inflict deleterious effects on EGFR-expressing healthy tissues, would exacerbate the side effects and limit the therapeutic window of EGFR inhibitors even further. On the contrary, innate cell engagers, such as AFM24, show no effect on the regenerative capacity of healthy tissue and are only active in tissue areas with high immune cell content and pro-inflammatory milieu. Therefore, the combinations involving innate cell engagers can be more efficacious due to benefit from a further immune cell activation, without an increase in on-target off-tumor toxicity.

One promising approach for the enhancement of innate cell engager efficacy targeting EGFR is to combine these agents with allogeneic or autologous NK cell products in order to increase the proportion of effector cell/tumor cell pairings following the trafficking and homing of both therapeutic modalities to tumor tissue. To achieve this, NK cells can either be pre-complexed with innate cell engagers prior to administration or both therapies can be co-administered separately. This approach has recently been shown to be successful in improving the antitumor activity of AFM13, an innate cell engager targeting the cell-surface CD30, in preclinical CD30^+^ Hodgkin lymphoma models (243). This approach is being investigated in a clinical proof-of-concept study with CD30^+^ Hodgkin and Non-Hodgkin patients (NCT04074746) (244). To investigate the combination of AFM24 with NK cells and to provide further evidence of such a combination strategy, a clinical study has recently been initiated that combines AFM24 with an autologous NK cell product (NCT05099549) (245). Such combinations may overcome the limitation of sparse distribution of effector cells in tumor tissue and may lead to improved effectiveness of both treatments, without potentially introducing additive adverse events (246, 247).

Another approach that may potentially enhance the effectiveness of innate cell engagers targeting EGFR is their combination with CPIs, such as anti-PD-1 or anti-PD-L1 antibodies. CPIs act to enhance immune responses and to inhibit the tumor escape from immunosurveillance by preventing the tumor cell-induced T cell and NK cell suppression (248). The combination of AFM13 with anti-PD1 has demonstrated high response rates in HL (249). Recent studies provided evidence for PD-1/PD-L1 expression not only on T cells but also on NK cells, which suggests a new level of mechanistic complexity behind diminished anti-tumor NK cell responses (175, 249, 250). In support of these findings, PD-L1 engagement by atezolizumab was shown to directly activate NK cell functions (250). A phase I/IIa study of AFM24 in combination with atezolizumab has recently been initiated (NCT05109442) (251).

The excellent safety profile of innate cell engagers may allow to exploit the combinations involving multiple therapeutic agents with different mechanisms of action to fully leverage a targeted anti-tumor immune system.

## Conclusions

EGFR has emerged as an oncogenic driver in a subset of patients with NSCLC, while widespread overexpression of EGFR protein has been found in a broad range of different types of cancer. Several different EGFR-targeting therapies have been developed and have entered the clinic but despite this, long-term survival rates of patients treated with these therapies have not significantly improved. Thus, there is a significant unmet need for therapies that are effective and safe in patients with EGFR-expressing solid tumors and can overcome currently documented inherent or acquired tumor resistance mechanisms to therapies targeting EGFR.

The response to TKIs in a subset of patients with NSCLC, whose tumors express EGFR carrying activating mutations, has been excellent but short-lived due to the inevitable evolution of cancer cells to acquire secondary resistance mutations, preventing the binding of TKIs to EGFR. Genetic alterations that activate signaling molecules downstream of EGFR or those in parallel signaling pathways can also create conditions where EGFR signaling, initially critical to the propagation of a tumor, is no longer required. Other EGFR-signaling inhibitors, such as anti-EGFR antibodies, have been approved in a subset of patients with *KRAS* wild-type mCRC, SCCHN and squamous NSCLC, but response rates are usually low and secondary resistance also develops through the redundancy of the EGFR signaling pathway or tumor transformation into another histologic type. On-target off-tumor and off-target toxicities to EGFR signaling inhibitors are of concern and may have a significant impact on patient quality of life and treatment effectiveness.

Overexpression of EGFR in many tumor types provides a basis for the design of therapies that use EGFR as a decoy to guide effectors of the adaptive or innate immune systems to EGFR-expressing cancers and to destroy EGFR-expressing cancer cells. Despite the promise of CAR-T cell therapies using cytotoxic T cells engineered to express constructs recognizing EGFR, these are still in early stages of development and present several serious complications that are currently difficult to overcome. Despite being effective in patients with hematologic malignancies, CAR-T cell therapies have not performed well in patients with solid tumors, potentially due to the hostile immunosuppressive tumor microenvironment and heterogeneity of cancer epitope expression. Serious neurotoxicity and CRS currently compromise the clinical use of these therapeutic agents.

To overcome the limitations of the T cell-based therapeutic approaches, target-specific innate cell engagers are undergoing preclinical and clinical evaluation and show high promise, in controlling on-target off-tumor toxicities. Based on preclinical investigations, the EGFR-specific innate immune system engager AFM24 activates potent mutation-independent antitumor immune responses by engaging NK cells and macrophages to mediate ADCC and ADCP and has shown a comparable activity to those therapies that utilize T cells. Importantly, the key advantage over therapies targeting EGFR signaling and those that use T cells is a much more favorable toxicity profile, with the lack of dermatologic toxicities and CRS, potentially determined by the lower proliferative potential of NK cells and macrophages and the EGFR signaling-independent mode of action of AFM24. These properties are key for delivering safer and more effective therapies to target tumors expressing EGFR. They also highlight the advantages of harnessing the innate immune system to address unmet needs in the treatment of patients with EGFR-expressing cancer.

## Author contributions

GH, ER, and HD designed, wrote, reviewed, revised, and edited the manuscript. All authors contributed to the article and approved the submitted version.

## Funding

This study received funding from Affimed GmbH.

## Acknowledgments

Medical writing assistance was provided by Meridian HealthComms, Plumley, UK and was funded by Affimed GmbH, in accordance with Good Publication Practice (GPP3).

## Conflict of interest

All authors are employees of Affimed GmbH.

The authors declare that this study received funding from Affimed GmbH. The funder had the following involvement with the study: funded the writing assistance.

## Publisher’s note

All claims expressed in this article are solely those of the authors and do not necessarily represent those of their affiliated organizations, or those of the publisher, the editors and the reviewers. Any product that may be evaluated in this article, or claim that may be made by its manufacturer, is not guaranteed or endorsed by the publisher.
